# Protocadherin-7 Regulates Osteoclast Differentiation through Intracellular SET-Binding Domain-Mediated RhoA and Rac1 Activation

**DOI:** 10.3390/ijms222313117

**Published:** 2021-12-04

**Authors:** Hyunsoo Kim, Noriko Takegahara, Yongwon Choi

**Affiliations:** Department of Pathology and Laboratory Medicine, Perelman School of Medicine, University of Pennsylvania, Philadelphia, PA 19104, USA; hyunsoo3@pennmedicine.upenn.edu (H.K.); tnoriko@pennmedicine.upenn.edu (N.T.)

**Keywords:** osteoclast, Pcdh7, RhoA, Rac1, SET, differentiation

## Abstract

Protocadherin-7 (Pcdh7) is a member of the non-clustered protocadherin δ1 subgroup of the cadherin superfamily. Although the cell-intrinsic role of Pcdh7 in osteoclast differentiation has been demonstrated, the molecular mechanisms of Pcdh7 regulating osteoclast differentiation remain to be determined. Here, we demonstrate that Pcdh7 contributes to osteoclast differentiation by regulating small GTPases, RhoA and Rac1, through its SET oncoprotein binding domain. Pcdh7 is associated with SET along with RhoA and Rac1 during osteoclast differentiation. Pcdh7-deficient (Pcdh7^−/−^) cells showed abolished RANKL-induced RhoA and Rac1 activation, and impaired osteoclast differentiation. Impaired osteoclast differentiation in Pcdh7^−/−^ cells was restored by retroviral transduction of full-length Pcdh7 but not by a Pcdh7 mutant that lacks SET binding domain. The direct crosslink of the Pcdh7 intracellular region induced the activation of RhoA and Rac1, which was not observed when Pcdh7 lacks the SET binding domain. Additionally, retroviral transduction of the constitutively active form of RhoA and Rac1 completely restored the impaired osteoclast differentiation in Pcdh7^−/−^ cells. Collectively, these results demonstrate that Pcdh7 controls osteoclast differentiation by regulating RhoA and Rac1 activation through the SET binding domain.

## 1. Introduction

Protocadherin-7 (Pcdh7) is a member of the non-clustered protocadherins, which comprise another phylogenetically distinct group of the cadherin superfamily [1]. Pcdh7 was originally identified from Xenopus embryo, termed NF-protocadherin (NFPC), followed by the cloning of human ortholog PCDH7 from the human gastric adenocarcinoma cell [2,3]. So far, Pcdh7 has been shown to play important roles in a range of physiological and pathological processes, including ectodermal cell sorting and vertebrate neural tube formation, cell morphology, axonal elongation in retinal ganglion cells, and brain metastatic lung and breast cancers [4,5,6,7,8,9,10]. Pcdh7 encodes an integral membrane protein and is thought to function not only in cell–cell recognition and adhesion through homophilic binding [9], but also in the regulation of cell signaling through its interaction with intracellular signaling proteins such as oncoprotein SET/Template-activating factor 1 (TAF1) and protein phosphatase-1α (PP1α) through the cytoplasmic domain [6,8,11,12,13]. In fact, the interaction of Pcdh7 with SET, a known oncoprotein that participates in cancer progression, has been shown to play an important role in Pcdh7-induced lung cancer transformation and tumorigenesis [14].

Osteoclasts are specialized multinucleated giant cells that resorb bone [15,16]. These cells are hematopoietic in origin and are derived from myeloid precursors. Osteoclast differentiation is driven by stimulation with the osteoclast differentiation factor Receptor activator of nuclear factor-kB ligand (RANKL), which is mainly provided by osteoblasts and osteocytes [17,18,19,20]. It further requires cell surface molecules to mediate multistep processes including migration, cell adhesion, and multinucleation, which is itself a hallmark of osteoclast maturation [21]. Rearrangement of the actin cytoskeleton occurs during osteoclast differentiation and maturation [22,23,24]. The Rho family small GTPases including RhoA, Rac1, and Cdc42, are key regulators of actin dynamics and have critical roles in osteoclast biology [25,26].

Recently, the requirement of Pcdh7 in osteoclast differentiation has been demonstrated in vitro and in vivo by generating Pcdh7 gene deletion (Pcdh7^−/−^) mice: Pcdh7^−/−^ mice exhibited increased bone mass due to impaired osteoclast differentiation [27,28]. However, the molecular mechanisms underlying the function of Pcdh7 in osteoclast differentiation remain unclear. Here, we demonstrate that Pcdh7 contributes to osteoclast differentiation by the regulation of small GTPase RhoA and Rac1 through a cytoplasmic SET binding domain. We found that Pcdh7 interacts with SET along with RhoA and Rac1 in pre-osteoclasts. Pcdh7-deficiency resulted in impaired RANKL-induced activation of RhoA and Rac1; consequently, Pcdh7-deficient cells failed to become fully differentiated osteoclasts. Impaired osteoclast differentiation in Pcdh7^−/−^ cells was restored by retroviral transduction of full-length Pcdh7 but not by a Pcdh7 mutant that lacks a SET binding domain (Pcdh7ΔSET). The direct crosslink of the Pcdh7 full intracellular region but not SET binding domain-deletion mutant induced activation of RhoA and Rac1 in pre-osteoclasts. Taken together, our findings reveal the role of Pcdh7 as a signaling receptor for osteoclast differentiation.

## 2. Results

### 2.1. Pcdh7 Associates with SET and Small GTPases RhoA and Rac1 in Osteoclasts

**Figure 1:**
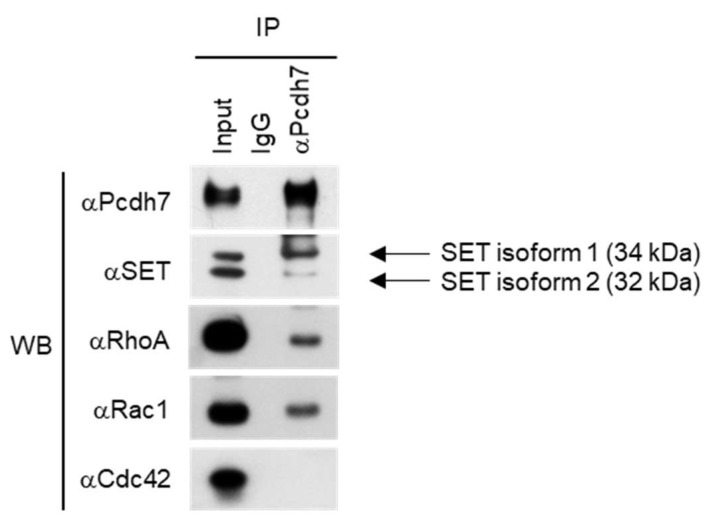
Pcdh7 associates with SET and small GTPase RhoA and Rac1. Total cell lysates were prepared from wild-type pre-osteoclasts cultured with M-CSF + RANKL for a day and used for immunoprecipitation (IP) and Western blotting (WB) with the indicated antibodies. IgG was used as a negative control antibody. Arrows indicate SET isoforms 1 and 2.

It has been reported that association with SET is important for the function of Pcdh7, prompting us to determine whether the association of Pcdh7 with SET is detected during osteoclast differentiation. Mouse bone marrow-derived monocytes (BMMs) were treated with M-CSF + RANKL for a day to induce pre-osteoclasts, then immunoprecipitation with anti-Pcdh7 antibody was performed, followed by Western blotting with anti-SET antibody. One day culture with RANKL induces pre-osteoclasts, which are committed to becoming fully mature multinucleated osteoclasts, and the expression levels of Pcdh7 protein peaked at this time [27]. The association of endogenous Pcdh7 with endogenous SET was detected (Figure 1). It has been reported that SET has two isoforms (SET1 and SET2) [29,30]. We detected both isoforms in pre-osteoclasts (Figure 1). Although the message expression of both isoforms was induced by stimulation with RANKL, which peaked at day 1 (Supplementary Materials), SET1 was the major isoform associated with Pcdh7 in pre-osteoclasts (Figure 1). SET has been shown to bind the Small GTPase Rac1 and is critical for Rac1-mediated signaling [31]. Therefore, we sought to determine whether small GTPases are involved in the molecular complex of Pcdh7 and SET. We examined the major small GTPases, RhoA, Rac1, and Cdc42. Pcdh7 is associated with RhoA and Rac1 but not Cdc42 (Figure 1). These results suggested the involvement of SET and small GTPases RhoA and Rac1 in Pcdh7-mediated osteoclast differentiation.

### 2.2. Pcdh7 Regulates Activation of Small GTPases RhoA and Rac1

**Figure 2:**
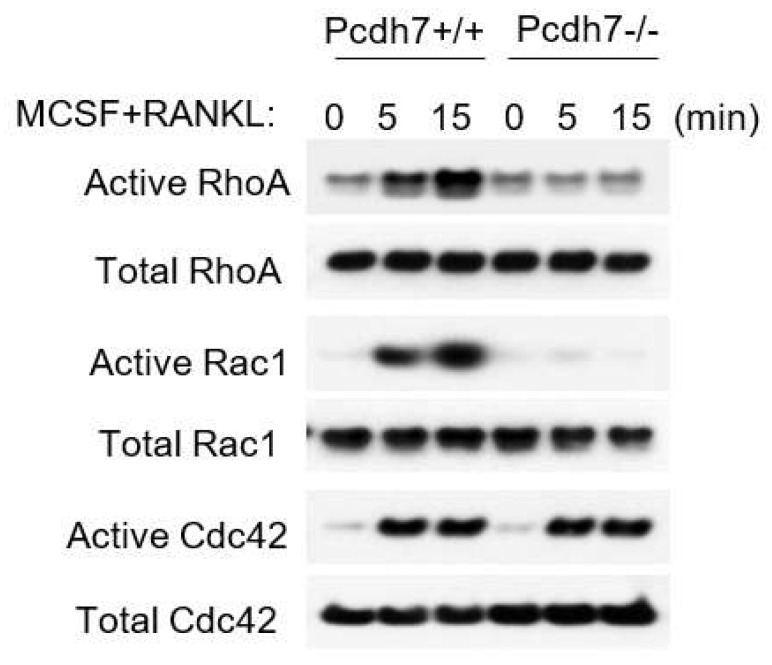
Pcdh7 is required for RhoA and Rac1 activation. Pre-osteoclasts were prepared from Pcdh7^+/+^ and Pcdh7^−/−^ BMMs cultured with M-CSF (60 ng/mL) + RANKL (150 ng/mL) for a day and stimulated with M-CSF + RANKL for the indicated times. The activated forms of RhoA, Rac1, and Cdc42 were detected by a pull-down assay.

To determine the involvement of RhoA and Rac1 in Pcdh7-mediated osteoclast differentiation, we examined the RANKL-induced activation of the small GTPases in the presence or absence of Pcdh7. Pre-osteoclasts were prepared from wild-type (Pcdh7^+/+^) and Pcdh7-deficient (Pcdh7^−/−^) mice and RANKL-induced activation was determined by a pull-down assay. Activation of RhoA, Rac1, and Cdc42 was observed in Pcdh7^+/+^ cells (Figure 2). By contrast, the activation of RhoA and Rac1 but not Cdc42 was abolished in Pcdh7^−/−^ cells (Figure 2). These results suggested that RhoA and Rac1 are involved in Pcdh7-mediated signaling during osteoclast differentiation.

### 2.3. SET-Binding Domain in Pcdh7 Intracellular Region Is Required for Pcdh7-Mediated Regulation of Osteoclast Differentiation

**Figure 3:**
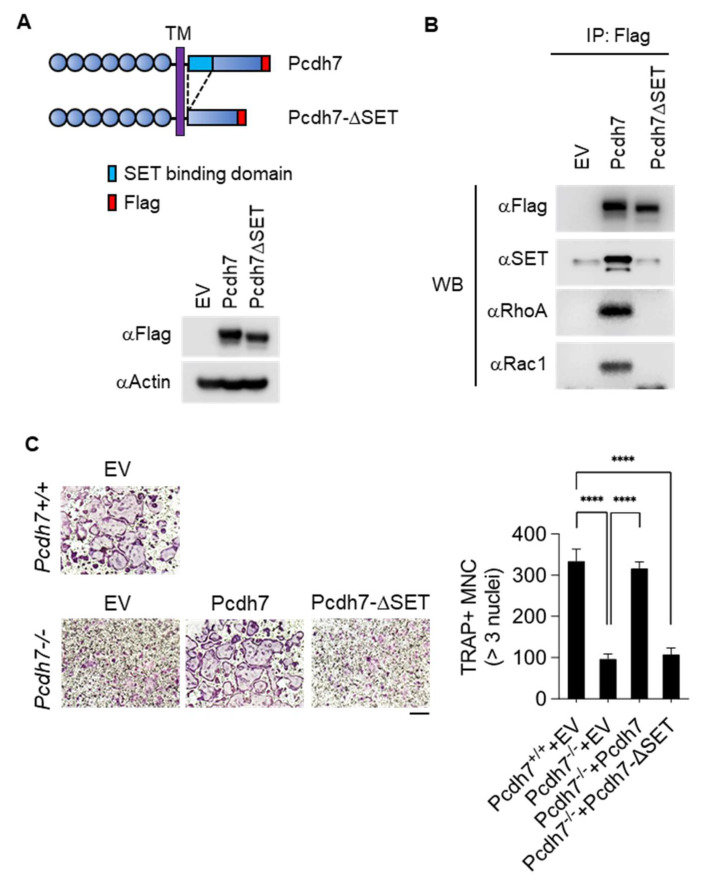
Pcdh7-deficiency is rescued by full-length Pcdh7 but not by Pcdh7ΔSET. (**A**). Schema of Pcdh7 wild-type and SET-binding domain deletion mutants (Pcdh7ΔSET). Both constructs were Flag-tagged at the C-terminus (top). Protein expression was confirmed by Western blotting with an anti-Flag antibody (bottom). (**B**). Coimmunoprecipitation of Pcdh7 with SET, RhoA, and Rac1. Pcdh7^−/−^ BMMs retrovirally transduced with the indicated vectors were lysed and immunoprecipitated with anti-Flag antibody, and Western blotting was performed with the indicated antibodies. (**C**). Osteoclast differentiation rescued by retroviral transduction of Pcdh7 but not by Pcdh7ΔSET in Pcdh7^−/−^ BMMs. BMMs were retrovirally transduced with empty vector (EV) or Flag-tagged Pcdh7 expression vectors followed by culture with M-CSF + RANKL for three days. The number of TRAP^+^ multinucleated cells (3 nuclei or more per cell) is shown. The scale bar represents 100 μm. Data are shown as the mean ± S.D. **** *p* < 0.0001.

To determine the involvement of SET in Pcdh7-mediated osteoclast differentiation, we generated an expression vector encoding a mutant Pcdh7 which lacks the first 67 amino acids of the intracellular region as the predicted SET-binding domain (Pcdh7ΔSET) (Figure 3A) [6]. Flag-tagged full-length Pcdh7 and Pcdh7ΔSET were retrovirally transduced into Pcdh7^−/−^ BMMs, and coimmunoprecipitation was performed to determine the association with SET and small GTPases RhoA and Rac1. As expected, no association of Pcdh7ΔSET with SET was observed (Figure 3B). The association of Pcdh7ΔSET with RhoA and Rac1 was not observed, either (Figure 3B). These results indicated that the SET-binding domain is required for the association of Pcdh7 with RhoA and Rac1. Retroviral transduction of full-length Pcdh7 fully restored the impaired formation of TRAP^+^ multinucleated osteoclasts in Pcdh7^−/−^ cultures, as we published previously (Figure 3C) [27]. By contrast, retroviral transduction of Pcdh7ΔSET failed to restore the formation of TRAP^+^ multinucleated osteoclasts (Figure 3C). These results suggested a critical role for the Pcdh7 SET-binding domain in Pcdh7-mediated osteoclast differentiation.

### 2.4. Crosslink of Pcdh7 Intracellular Region Is Critical for Activation of RhoA and Rac1, and Formation of Osteoclasts

**Figure 4:**
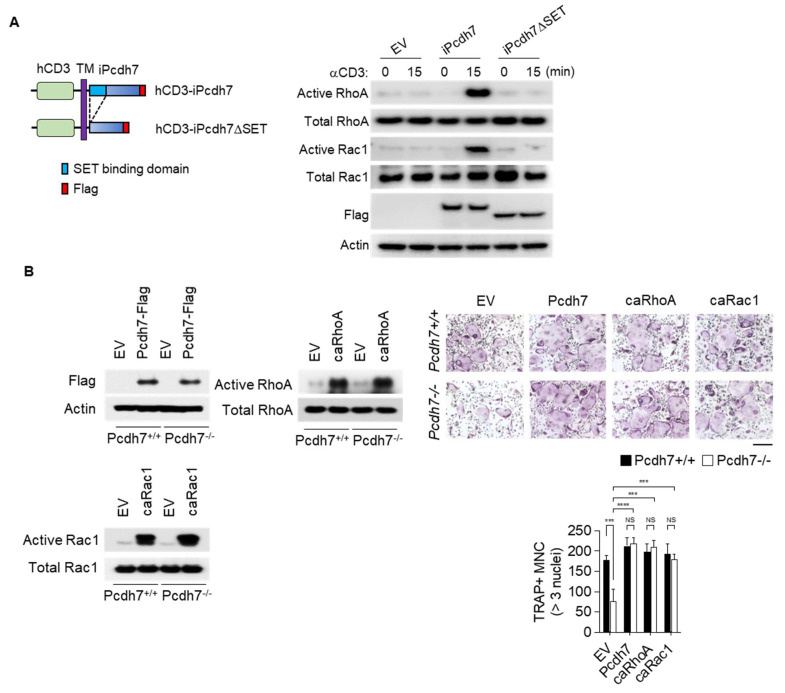
Crosslink of Pcdh7 intracellular region is critical for activation of RhoA and Rac1, and formation of osteoclasts. (**A**). Schema of human CD3 extracellular-transmembrane region fused to Pcdh7 full intracellular region (hCD3-iPcdh7) or Pcdh7ΔSET intracellular region (hCD3-iPcdh7ΔSET) chimeric proteins. Both constructs were Flag-tagged at C-terminus (left). Pcdh7^−/−^ BMMs retrovirally transduced with the indicated vectors were cultured with M-CSF + RANKL for a day and stimulated with anti-hCD3 antibody for 15 min. Total cell lysates were used to detect the activated forms of RhoA and Rac1 (right). (**B**). Pcdh7^+/+^ and Pcdh7^−/−^ BMMs retrovirally transduced with the indicated vectors were cultured with M-CSF + RANKL for 3 days to induce osteoclasts. Expression of transduced vectors was confirmed by Western blotting (left). The number of TRAP^+^ multinucleated cells (3 nuclei or more per cell) is shown (right). The scale bar represents 100 μm. Data are shown as the mean ± S.D. *** *p* < 0.001, **** *p* < 0.0001.

Having shown the involvement of RhoA and Rac1 in Pcdh7-mediated signaling and a critical role for the Pcdh7 SET-binding domain in Pcdh7-mediated osteoclast formation, we sought to further investigate the mechanisms by which Pcdh7 regulates osteoclast differentiation. To gain insight into the function of the Pcdh7 SET-binding domain during osteoclast differentiation, we employed an approach to directly induce the activation of the Pcdh7 cytoplasmic region specifically in a controlled manner, by generating expression vectors encoding chimeric proteins that fuse the human CD3 (hCD3) extracellular-transmembrane region to the Pcdh7 full intracellular region (hCD3-iPcdh7) or the Pcdh7ΔSET intracellular region (hCD3-iPcdh7ΔSET) (Figure 4A). Pcdh7^−/−^ BMMs retrovirally transduced with hCD3-iPcdh7 or hCD3-iPcdh7ΔSET were cultured with M-CSF + RANKL for a day, and stimulated with anti-hCD3 antibody to crosslink the surface hCD3 to induce stimulation of the Pcdh7 intracellular region. Activation of RhoA and Rac1 by crosslinking hCD3 was examined by a pull-down assay. Stimulation with anti-hCD3 antibody activated RhoA and Rac1 in hCD3-iPcdh7-expressing cells (Figure 4A). By contrast, anti-hCD3 antibody stimulation failed to activate these small GTPases in hCD3-iPcdh7ΔSET-expressing cells (Figure 4A). These results suggested that crosslinking the Pcdh7 intracellular region induces the activation of RhoA and Rac1 through the SET-binding domain.

We then sought to identify whether impaired activation of RhoA and Rac1 is the bottleneck hindering osteoclast differentiation in Pcdh7^−/−^ cells. The constitutive active form of RhoA (caRhoA) and Rac1 (caRac1) expression vectors were retrovirally transduced into Pcdh7^+/+^ and Pcdh7^−/−^ BMMs, and then cultured with M-CSF + RANKL for three days to induce fully differentiated osteoclasts. A full-length Pcdh7 expression vector was used as a positive control. Expression of caRhoA and caRac1 restored osteoclast differentiation in Pcdh7^−/−^ cells to the same degree as the full-length Pcdh7 expression vector (Figure 4B). Taken together, these results suggest that the crosslinking of the Pcdh7 intracellular region activates RhoA and Rac1 through its SET-binding domain, which is critical for osteoclast differentiation.

## 3. Discussion

**Figure 5:**
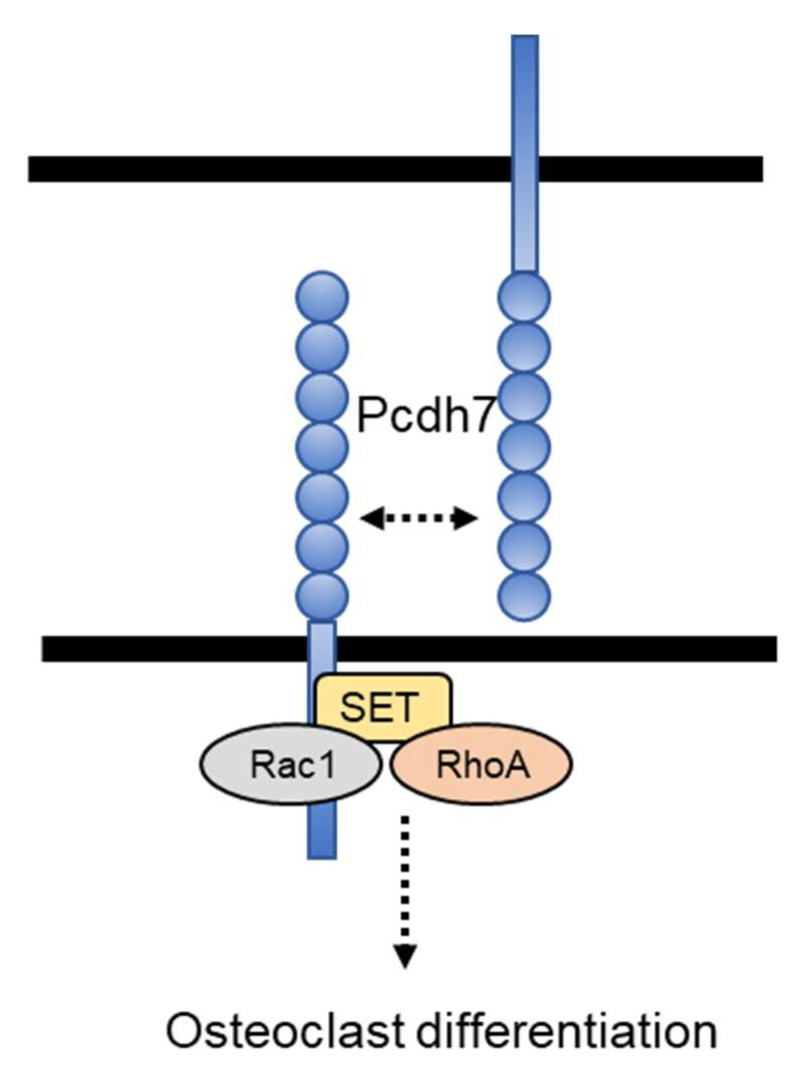
Schematic model of regulation of osteoclast differentiation by Pcdh7. Pcdh7 interacted with SET and small GTPases RhoA and Rac1 through the SET-binding domain. Ligation of Pcdh7, which might be mediated in a homophilic manner, activates intracellular signaling including RhoA and Rac1 activation and, consequently, induces osteoclast multinucleation and differentiation. Dashed two-way arrow indicates assumption. Dashed arrow presumes involvement of a number of signaling molecules.

In this study, we demonstrated that Pcdh7, a member of the non-clustered protocadherin δ1 subgroup of the cadherin superfamily, regulates osteoclast differentiation by inducing small GTPases RhoA and Rac1 activation. Additionally, we identified that an intracellular SET-binding domain is essential for RhoA and Rac1 activation (Figure 5). Complete restoration of the impaired differentiation of Pcdh7^−/−^ cells by overexpression of the constitutively active form of RhoA and Rac1 confirmed the importance of these small GTPases in Pcdh7-mediated osteoclast formation. Rac1 has been reported to be required for osteoclast generation in vivo [32], suggesting that the increased bone phenotype observed in Pcdh7^−/−^ is due in part to impaired Pcdh7-mediated Rac1 activation in osteoclasts. Our findings reported here provide evidence of the role of Pcdh7 as a signaling receptor for osteoclast differentiation.

Rho family GTPases perform several cellular functions in osteoclasts, the most prominent of which is regulating the dynamic actin rearrangements. Given that the differentiation/maturation of osteoclasts relies on the organization of the cytoskeleton, it is plausible that Pcdh7-mediated activation of RhoA and Rac1 is required for the regulation of actin dynamics during osteoclast differentiation. RhoA and Rac1 are known to be critical for osteoclast bone resorbing activities [33,34]. However, Pchd7-deficiency resulted in extremely small reduction, if any, of osteoclastic bone resorption [27], suggesting that Pcdh7-mediated activation of RhoA and Rac1 is not essential for bone resorbing function. Although we showed importance of Pcdh7-mediated activation of RhoA and Rac1 during osteoclast differentiation, antagonistic functions of Rho and Rac on spreading of multinucleated cells has also been reported [35]. These observations suggested complex and multiple functions of Rho and Rac in osteoclasts. Further studies will be required to clarify the regulatory mechanisms of small GTPases in the context of Pcdh7-mediated osteoclast differentiation. Rac1 is one of the essential components of NADPH oxidase, which is responsible for generating reactive oxygen species [36,37,38,39]. RANKL-induced generation of ROS has been shown to function as an intracellular signal mediator for osteoclast differentiation [40]. It remains to be determined whether Pcdh7-mediated Rac1 activation contributes to osteoclast formation through the generation of ROS.

Pcdh7 has been thought to function not only in cell adhesion through homophilic binding, but also in the regulation of cell signaling. By designing a chimeric receptor with the human CD3 extracellular domain and the Pcdh7 intracellular region, we could induce Pcdh7-dependent signals by crosslinking chimeric receptors using anti-human CD3 antibodies. These tools enabled us to activate Pcdh7-mediated signals in a controlled and synchronized manner. Through this approach, we showed that CD3-mediated receptor aggregation induced the activation of RhoA and Rac1. Our results demonstrated that Pcdh7-mediated intracellular signaling is critical for osteoclast differentiation. Pcdh7 has been reported to have a homophilic interaction in the retinal axon [9]. Given the osteoclast-intrinsic role of Pcdh7 [27], it is plausible that Pcdh7 interacts in a homophilic manner during osteoclast differentiation. Although we demonstrated the role of Pcdh7 as a mediator of cell signaling, we cannot exclude the possibility of it being a cell adhesion molecule during osteoclast differentiation. Cell–cell interactions mediated by cell surface receptors are required for osteoclast differentiation during which reorganization of cytoskeleton mediation by Rho and Rac occurs. Further studies are required to clarify the impact of Pcdh7-mediated cell signaling (including involvement of Pcdh7 in sub-cellular localization of osteoclast master regulators such as NFATc1 during osteoclast differentiation) and cell adhesion in the context of osteoclast differentiation.

We identified RANKL-induced expression of SET, which peaked on day 1. The message expression of SET (SET1 and SET2—both isoforms) was comparable between Pcdh7^+/+^ and Pcdh7^−/−^ cells, suggesting that the expression of SET isoforms is not perturbed by the absence of Pcdh7. We demonstrated that the SET-binding domain of the Pcdh7 intracellular region is required for RhoA and Rac1 activation. These findings suggest a role of SET in Pcdh7-mediated osteoclast differentiation. However, the principal function of SET in osteoclasts remains unknown. A study has reported that Pcdh7 recruits SET along with protein phosphatase 2A (PP2A), which is inhibited by SET, thereby suppressing PP2A activity [14]. PP2A has been shown to regulate RhoA in T cells [41], and Rac1 in lung cancer [42], implying SET-mediated regulation of PP2A in osteoclasts. Future studies will be required to understand the molecular function of SET in Pcdh7-mediated regulation of osteoclast differentiation. We cannot exclude the possibility of requirement of additional Pcdh7 intracellular region for osteoclast differentiation, and further studies will be required to address this issue.

Taken together, we provide evidence here that Pcdh7 regulates RhoA and Rac1 activities through the intracellular SET binding domain, contributing to RANKL-induced osteoclast differentiation.

## 4. Materials and Methods

### 4.1. In Vitro Cell Culture, Osteoclast Differentiation, and Tartrate-Resistant Acid Phosphatase (TRAP) Staining

BMMs and osteoclasts from wild-type (Pcdh7^+/+^) and Pcdh7^−/−^ mice were prepared, as described previously [43]. In brief, whole bone marrow was extracted from the femurs and tibias of mice and incubated in 100 mm Petri dishes in α-MEM medium containing 10% fetal bovine serum and M-CSF (5 ng/mL) overnight. Non-adherent cells were collected and cultured for three days with M-CSF (60 ng/mL) to generate BMMs. For OC differentiation, BMMs were plated at 5 × 10^3^ per well in 96-well cell culture plates or 5 × 10^5^ per well in 6-well cell culture plates and cultured with M-CSF (60 ng/mL) and RANKL (150 ng/mL) for 3 days. Osteoclasts were stained using Leukocyte acid phosphatase Kit (387A-1KT, Sigma-Aldrich, St Louis, MO, USA) following the manufacturer’s instructions. Anti-human CD3 (HIT3a) antibodies were purchased from Biolegend (Cat.# 300302, San Diego, CA, USA).

### 4.2. Coimmunoprecipitation, Pull-Down Assay, and Western Blotting

For coimmunoprecipitation, cultured cells were lysed with IP lysis buffer (25 mM Tris-HCl pH 7.4, 150 mM NaCl, 1 mM EDTA, 1% NP-40, and 5% glycerol, protease inhibitor cocktail (Roche, Indianapolis, IN, USA)). The lysates (500 μg–1 mg of protein) were coimmunoprecipitated overnight using antibodies, at 4 °C. For small-GTPase activity, RhoA, Rac1, and Cdc42 assays were conducted using a RhoA/Rac1/Cdc42 Activation Assay Combo Biochem kit (Cat.# BK030, Cytoskeleton, Inc., Denver, CO, USA) according to the manufacturer’s instructions. For Western blotting, cell cultures in 6-well plates were washed with ice-cold phosphate-buffered saline (PBS) and lysed with ice-cold radio immunoprecipitation (RIPA) lysis buffer (20 mM Tris-HCl, pH 7.5, 150 mM NaCl, 1% NP-40, 0.5% sodium deoxycholate, 1 mM EDTA, 0.1% SDS, protease and phosphatase inhibitor cocktail). The lysates were centrifuged to remove debris and protein concentrations were determined using the Bradford assay. Equal amounts of lysates (50–100 μg of protein) were fractionated by SDS-polyacrylamide gel electrophoresis (SDS-PAGE) on 4%–12% gradient gels and transferred onto polyvinyl difluoride (PVDF) membranes. Western blotting was performed with the following antibodies: anti-Pchd7 (Cat.#139274, Abcam, Cambridge, MA, USA), anti-SET (Cat.#181990, Abcam, Cambridge, MA, USA), anti-RhoA (Cat.#ARH04, Cytoskeleton, Inc, Denver, CO, USA), anti-Rac1 (Cat.#ARC03, Cytoskeleton, Inc, Denver, CO, USA), anti-Cdc42 (Cat.#ACD03, Cytoskeleton, Inc, Denver, CO, USA.), anti-Flag: M2 (Sigma-Aldrich, St Louis, MO, USA) and anti-actin (Cat.#sc-47778, Santacruz Biotechnology, SantaCruz, CA, USA).

### 4.3. Generation of Constructs

The pMX vector encoding C-terminally FLAG-tagged full-length mouse Pcdh7 (pMX-Pcdh7) was used previously [27]. Mouse Pcdh7 SET binding domain-deletion intracellular region (iPcdh7ΔSET) was amplified by PCR with the following primers: a sense primer with the NsiI site (5′-TTTATGCATGTCAGACAGCCCAAGCATGGGCCGA-3′) and an antisense primer with the XhoI site (5′-TTTCTCGAGCAGATAAACTTCTCTTCTAGTGAG-3′) and cloned into the PstI-XhoI fragment of the pCMV6 vector encoding mouse Pcdh7. Then, BamHI-XhoI fragment of the mouse Pcdh7ΔSET was cut out from the vector and subcloned into the BamHI-XhoI fragment of the pMX-Flag vector to generate the pMX-Pcdh7ΔSET vector. Mouse Pcdh7 intracellular region (iPcdh7) and ΔSET intracellular region (iPcdh7ΔSET) were amplified with the following primers: sense primers with the SalI site (5′-TTTGTCGACTGCAGGT CCAAAAATAA AAATGGC-3′ for iPcdh7, and 5′-TTTGTCGACTCAGACAGCCCAAGCATGGGCCGA-3′ for iPcdh7ΔSET) and an antisense primer with the XhoI site (5′-TTTCTCGAGCAGATAAACTTCTCTTCTAGTGAG-3′) and cloned into the XhoI fragment of the pMX-hCD3-Flag vector [44] to generate pMX-hCD3-iPcdh7 vector and pMX-hCD3-iPcdh7ΔSET vector.

### 4.4. Retrovirus Preparation and Transduction

To prepare retroviral particles, Plat-E packaging cells were plated on 100mm culture dishes and transfected pMX-Pcdh7, pMX-Pcdh7ΔSET, pMX-hCD3-iPcdh7, and pMX-hCD3-iPcdh7ΔSET using PEImax (Polysciences, Warrington, PA, USA). Empty pMX vector was used as a negative control. After 3 days, the medium containing the retrovirus was harvested and passed through a syringe filter (0.45 μm pore diameter). BMMs were transduced with retroviruses for 16 h with hexadimethrine bromide (8 μg/mL) in the presence of M-CSF (60 ng/mL). After washing with fresh medium, infected cells were selected by culturing for 2 days in the presence of puromycin (2 μg/mL) with M-CSF (60 ng/mL). Puromycin-resistant BMMs were used for experiments.

### 4.5. Statistical Analysis

All experiments were analyzed using one-way ANOVA or two-way ANOVA by Prism 7.0 (GraphPad Software Inc, San Diego, CA, USA). *p* < 0.05 was considered statistically significant.

## Supplementary Materials

The following are available online at https://www.mdpi.com/article/10.3390/ijms222313117/s1.
