# Supplementary material for: Protocadherin-7 Regulates Osteoclast Differentiation through Intracellular SET-Binding Domain-Mediated RhoA and Rac1 Activation
Source: Int J Mol Sci. 2021 Dec 4;22(23):13117. doi: 10.3390/ijms222313117 (PMC8658210; doi:10.3390/ijms222313117)
Supplement: Supplementary file 1 [file ijms-22-13117-s001.zip › IJMS_Pcdh7 supplementary information.pdf]

Materials and methods S1

Reverse transcription and real-time PCR (Q-PCR)

Total RNAs were extracted from cells using Trizol reagent (Invitrogen), and 1–5 μg of total RNA was reverse-transcribed using random hexamer primers and SuperScript III reverse transcriptase (Invitrogen). cDNA corresponding to 10 ng of total RNA was analyzed by Q-PCR using a QuantStudio3 (Applied Biosystems). Q-PCR was performed through SYBR green (Applied Biosystems). The sequences of the primers for SET1 and SET2 are as follows: SET1 (forward: AGTCTGCGATCCTGCCTCAG, reverse: AGCAGTGCAGACACTTGTGG), SET2 (forward: GCTCAACTCCAATCACGACG, reverse: AGCAGTGCAGACACTTGTGG). Mouse 18S primers (forward: GCCATGCATGTCTAAGTACGC, reverse: TCTGATAAATGCACGCATCC) were used for normalization.

Supplemental S1 legend

Expression profiles of SET isoforms 1 and 2 during osteoclast differentiation. Total RNA was collected from Pcdh7$^{+/+}$ and Pcdh7$^{-/-}$ cultures and the levels of the indicated genes were measured by Q-PCR.
