# Supplementary figures and images for: Protocadherin-7 Regulates Osteoclast Differentiation through Intracellular SET-Binding Domain-Mediated RhoA and Rac1 Activation
Source: Int J Mol Sci. 2021 Dec 4;22(23):13117. doi: 10.3390/ijms222313117 (PMC8658210; doi:10.3390/ijms222313117)

## Supplementary figure

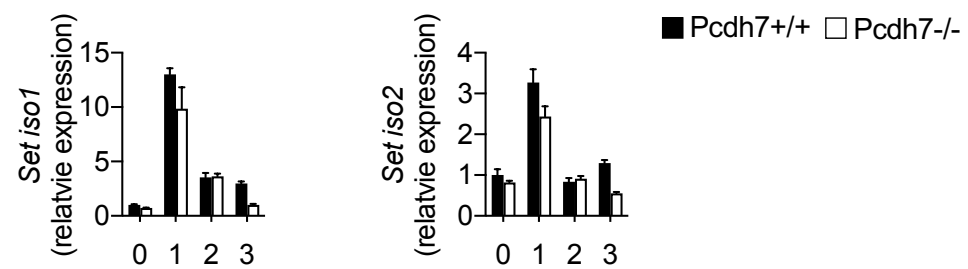

Supplement: Supplementary file 1 [file ijms-22-13117-s001.zip › IJMS_Pcdh7 supplementary figure.pdf]
